# Engineered apoptotic bodies hitchhiking across the blood-brain barrier achieved a combined photothermal-chemotherapeutic effect against glioma

**DOI:** 10.7150/thno.80632

**Published:** 2023-05-15

**Authors:** Yu Liu, Dehong Hu, Duyang Gao, Ping Gong, Hairong Zheng, Minjie Sun, Zonghai Sheng

**Affiliations:** 1 Paul C. Lauterbur Research Center for Biomedical Imaging, Institute of Biomedical and Health Engineering, Shenzhen Institute of Advanced Technology, Chinese Academy of Sciences, Shenzhen, 518055, P.R. China.; 2 State Key Laboratory of Natural Medicines, Department of Pharmaceutics, China Pharmaceutical University, Nanjing, 210009, P. R. China.; 3 Guangdong Key Laboratory of Nanomedicine, CAS Key Laboratory of Health Informatics, Shenzhen Bioactive Materials Engineering Lab for Medicine, Institute of Biomedicine and Biotechnology, Shenzhen Institute of Advanced Technology, Chinese Academy of Sciences, Shenzhen, 518055, P. R. China.; 4 Cancer Centre, Institute of Translational Medicine, Faculty of Health Sciences, University of Macau, Taipa, Macau SAR, 999078, P. R. China.

**Keywords:** Apoptotic bodies, Blood-brain barrier, Drug delivery, Glioma, Photothermal therapy

## Abstract

**Background:** Glioma as a highly lethal tumor is difficult to treat since the blood-brain barrier (BBB) restricts drug delivery into the brain. It remains a huge need for developing strategies allowing drug passage across the BBB with high efficacy.

**Methods:** Herein, we engineered drug-loaded apoptotic bodies (Abs) loaded with doxorubicin (Dox) and indocyanine green (ICG) to cross the BBB for the treatment of glioma. The confocal laser scanning microscopy was used to characterize the structure and evaluate the hitchhiking effect of the Abs. The *in vivo* BBB-crossing ability and photothermal-chemotherapeutic effect of the drug-loaded Abs were investigated in mice orthotopic glioma model.

**Results:** Engineered Abs loaded with Dox and ICG were successfully prepared. The Abs were phagocytized by macrophages, actively penetrate the BBB *in vitro* and* in vivo* utilizing the hitchhiking effect. The whole *in vivo* process was visualized by near-infrared fluorescence signal with a signal-to-background ratio of 7 in a mouse model of orthotopic glioma. The engineered Abs achieved a combined photothermal-chemotherapeutic effect, leading to a median survival time of 33 days in glioma-bearing mice compared to 22 days in the control group.

**Conclusions:** This study presents engineered drug carriers with the ability to hitchhike across the BBB, providing new opportunities for the treatment of glioma.

## Introduction

Glioma is a highly lethal tumor that starts in the glial cells of the brain or the spinal cord 1-3. Surgical resection is the most effective intervention, but glioma is difficult to treat due to its rapid progression and blurred tumor margins 4, 5. Other interventions, such as chemotherapy 6-8 and phototherapy 9, 10 are used to enhance the therapeutic efficacy. The blood-brain barrier (BBB) limits the passage of chemotherapeutic drugs or photosensitizers into the brain, reducing their potential benefits 11-13. There is a current need for developing more efficient drug delivery strategies allowing drug passage across the BBB 14, 15.

Novel procedures have been implemented to deliver drugs into the brain, such as surface modification 16, 17, bionic 18-21 and cell-based drug delivery systems (DDSs) 22-24. Cell-based DDSs combine the physiological properties (e.g., the low immunogenicity and tumor-homing ability) of endogenous cells 25, 26 with the physical and chemical characteristics (e.g., the high drug-loading efficiency and ease of modification) of synthetic nanocarriers 27, 28. Xue et al. reported that neutrophils carrying liposomal paclitaxel crossed the BBB and suppressed the recurrence of glioma efficiently 29. However, cell-based DDSs constructed before *in vivo* administration usually suffer from reduced cell viability, premature leakage of drugs and low possibility of large-scale preparation 30, 31.

Apoptotic bodies (Abs) are membrane vesicles released from fragmented apoptotic cells 32-34. Recently, they have been used as an alternative DDS for the treatment of cancer and inflammatory disorders 35-38. Abs are convenient to obtain at relatively low prices and are efficiently produced. The high expression of phosphatidylserine (PS) on the surface of Abs can be an “eat-me” signal, making it easy for Abs to be phagocytosed by monocytes/macrophages (designated MAs). The MAs can recognize chemokines released by tumor cells and home to tumor sites, thereby delivering drugs to tumor sites. This targeted delivery of drugs that relies on the intrinsic homing properties of endogenous cells is known as the hitchhiking effect 31, 39. Zheng et al. described an ultraviolet irradiation method to load Abs with gold nanorods and immune adjuvant unmethylated cytosine-guanine dinucleotide (CpG) as photothermal immunotherapy against cancer 39. Despite some positive results, the use of ultraviolet light to produce drug-loaded Abs was described as inefficient, requiring additional drug-loading processes 40-42. Whether the Abs can cross the BBB and target glioma tumor tissues have not yet been evaluated.

**Figure 1:**
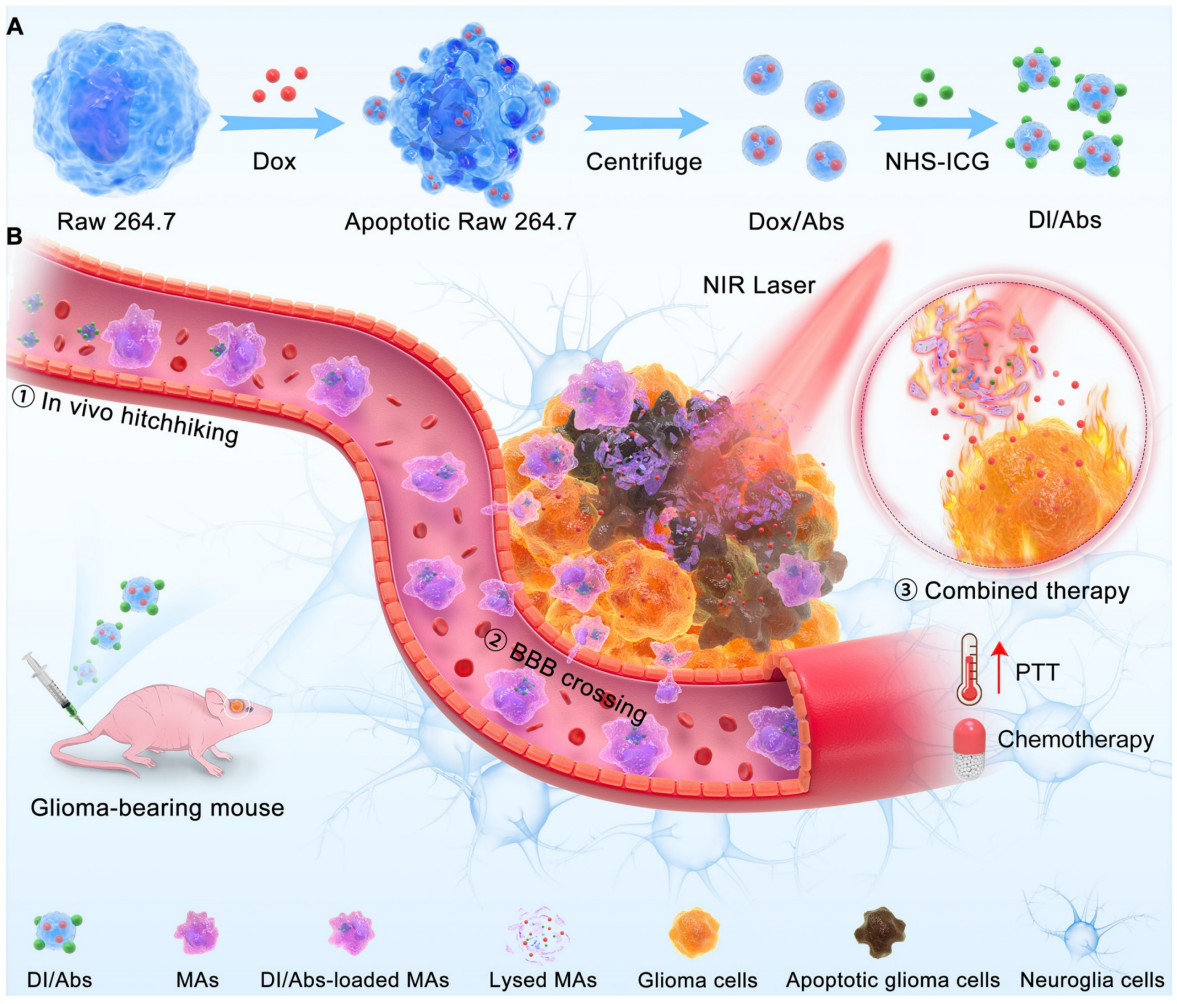
Schematic illustration of (**A**) preparation of DI/Abs and (**B**) the mechanism of DI/Abs hitchhiking across BBB and the combined photothermal-chemotherapeutic effect against glioma.

Herein, we reported an engineered method to obtain Abs loaded with doxorubicin (Dox) and indocyanine green (ICG) (Figure 1A). These Abs, defined as DI/Abs, can be specifically phagocytosed by monocytes/macrophages in the blood. Under the action of the chemokine released by tumor cells, monocytes/macrophages carrying DI/Abs pass through the tight junction layer of cerebrovascular endothelial cells through “deformation” and enter the tumor tissue 43, 44. The bright near-infrared fluorescence of DI/Abs allowed us to visualize the hitchhiking process. The photothermal effect of DI/Abs eliminated tumor cells and promoted the release of Dox to kill residual tumor cells, achieving a combined photothermal-chemotherapeutic effect against glioma (Figure 1B). These findings showed the potential for DI/Abs as an efficient DDS for the treatment of glioma.

## Methods and Materials

### Materials

Doxorubicin hydrochloride was obtained from Meilunbio (Dalian, China). ICG was bought from InnoChem (United States). NHS-ICG was bought from BioActs (South Korea). Annexin V-PE was obtained from MBL (Beijing, China). Hoechst 33342, DAPI, calcein AM/propidium iodide double stain kit, Cell Counting Kit-8 (CCK-8) and Western-IP cell lysis buffer were bought from Beyotime (Shanghai, China). The cell culture components were obtained from Gibco (United States). The H&E staining kit, F4/80 antibodies and Cy3-labeled goat anti-rabbit IgGs were obtained from Servicebio (Wuhan, China).

### Cell culture

Raw 264.7 cells, bEnd.3 endothelial cells, C6 glioma cells and luciferase-labeled C6 glioma cells (C6-Luc) were obtained from the American Type Culture Collection. The Dulbecco's modified Eagle's medium (DMEM) was used as the culturing medium. The fetal bovine serum and streptomycin/penicillin were added to DMEM at 10% and 1% concentration, respectively.

### Preparation and characterization of DI/Abs

Dox (20 μg/mL) was added to Raw 264.7 cells (1~2 × 10^7^) and incubated at 37℃ for 12 h to induce apoptosis. Then, the medium was harvested and centrifuged at 300 g for 10 min. We discarded apoptotic cells and cell debris precipitated at the bottom. The supernatant was further centrifuged at 3000 g for 30 min and washed twice with PBS to remove free Dox to obtain Dox/Abs. The Dox/Abs were resuspended in PBS, reacted with NHS-ICG in the dark for 1 h, washed twice with PBS to remove unreacted NHS-ICG to obtain the final DI/Abs. The particle size of DI/Abs was detected by Zetasizer Nano ZS (Malvern, United Kingdom). The morphology of DI/Abs was observed through a scanning electron microscope (Hitachi, Japan). The ultraviolet/visible/near-infrared absorption and fluorescence spectra of DI/Abs were obtained from an ultraviolet/visible/near-infrared spectrometer (Lambda 25, PerkinElmer, United States) and a fluorescence spectrophotometer (F900, Edinburgh Instruments, Ltd., United Kingdom), respectively. In order to measure the drug loading in DI/Abs, the number of Abs was firstly measured using a particle counter (PSS AccuSizer 780A, United States), and then DI/Abs were lysed with Western-IP lysate, The ICG and Dox loading efficacy in apoptotic bodies can be measured by the absorption spectrum of ICG and the fluorescence spectrum of Dox, respectively. To confirm that the engineered DI/Abs were successfully loaded with Dox and ICG, the exposed PS was stained with Annexin V-PE and the fluorescence signal distribution of Dox, ICG and Annexin V-PE was observed by confocal laser scanning microscopy (Zeiss, Germany). The temperature change was recorded after PBS, free ICG, ICG/Abs and DI/Abs were irradiated with laser (wavelength = 808 nm, power density = 1 W/cm^2^) for 5 min.

### Preparation of ICG/Abs

Raw 264.7 cells were irradiated with ultraviolet light (0.1 J/cm^2^) for 30 min and incubated for 12 h. The medium was harvested and centrifuged at 300 g for 10 min, and apoptotic cells and cell debris precipitated at the bottom were discarded. The supernatant was further centrifuged at 3000 g for 30 min to obtain blank Abs. The Abs were resuspended with PBS and NHS-ICG was added for incubation for 1 h to obtain the final ICG/Abs.

### Cytotoxicity assay

Raw 264.7 cells (1 × 10^4^/well) were placed in a 96-well plate. Then, the medium was replaced with another medium containing DI/Abs at different concentrations and cells were incubated for 12 h. The CCK-8 assay measured the viability of Raw 264.7 cells.

### Cellular uptake assay

Raw 264.7 cells (5 × 10^4^) were placed in a 35-mm confocal petri dish and incubated for 12 h. Then, free ICG, free Dox, the mixture of ICG and Dox and DI/Abs (C_ICG_ = 10 μg/mL, C_Dox_ = 5 μg/mL) were added and cells were incubated for another 2 h. After staining with Hoechst33342, cells were imaged with confocal laser scanning microscopy. The uptake of ICG by DI/Abs or free ICG treated-Raw 264.7 cells was measured by using flow cytometry (APC chanel, excitation wavelength = 633 nm, emission wavelength = 660 nm).

### Effect of different ICG concentration modifications on the uptake of Abs

We used different concentrations of ICG on the surface of Abs with a fixed concentration of Dox. Abs with different ICG/Dox ratios (from 0 to 4) were added to Raw 264.7 cells and incubated for 2 h. The cells were washed, fixed and stained with Hoechst33342. The uptake of Abs by Raw 264.7 cells was observed by using confocal laser scanning microscopy.

### The BBB model *in vitro*

The bEnd.3 cells were placed into the upper chamber pre-coated with gelatin to form cell monolayers. The cell medium was added and changed every other day until cells formed tight junctions. The transepithelial electrical resistance was measured using a CellZscope system (NanoAnalytics, Germany) to evaluate the suitability of the model. Then, a number of C6 cells were placed into the lower chamber to simulate a glioma environment. The separate DI/Abs (group Ⅰ) or DI/Abs-loaded Raw 264.7 cells (group Ⅱ) were later added to the upper chamber. The group III was investigated with DI/Abs-loaded Raw 264.7 cells in the upper chamber and without C6 cells in the lower chamber. After 4 h of incubation, the cells and mediums of upper chamber, lower chamber and bEnd.3 cell layers were harvested. The content of Dox was measured by high-performance liquid chromatography.

### Photothermal effect promoting Dox release and transport to C6 glioma cells

Raw 264.7 cells (5 × 10^4^/well) were incubated in a 24-well plate overnight. Then, a specific amount of DI/Abs was added. After 4 h incubation, cells were washed twice, cultured in PBS and divided into two groups: one group was irradiated with laser (wavelength: 808 nm, power density: 1 W/cm^2^) for 5 min to fully lyse the Raw 264.7 cells, the other group was not treated. After an additional 12 h incubation, the content of Dox in the supernatant and adherent cells was measured in each group. To further verify whether released Dox from Raw 264.7 cells entered in C6 glioma cells, laser-irradiated and untreated DI/Abs-loaded Raw 264.7 cells were co-incubated with C6 glioma cells for 12 h, washed and fixed. The content of Dox in C6 glioma cells was observed by confocal laser scanning microscopy.

### Toxicity of released Dox in C6 cells

C6 cells were incubated in a 96-well plate overnight to investigate the toxicity of released Dox. The supernatant from DI/Abs-loaded Raw 264.7 cells with/without near-infrared laser irradiation was added to the medium of C6 cells. After 24 h incubation, the viability of C6 cells was measured by the CCK-8 assay. The C6 cells were further treated with supernatants from different treatment groups for 24 h. The C6 cells were died with calcein-AM and propidium iodide and imaged with fluorescence microscopy.

### The mouse model of orthotopic glioma

Female Balb/c nude mice and Balb/c mice weighing 18-22 g were acquired from Beijing Vital River Laboratory Animal Technology Co., Ltd. (China). All animal experiments were approved by the Animal Care and Use Committee of Shenzhen Institutes of Advanced Technology, Chinese Academy of Sciences (China). The mouse model of orthotopic glioma was constructed according to previously published studies 18. Briefly, Balb/c nude mice were immobilized and anesthetized. Then, a small hole was drilled on the right side of the bregma. Five microliters of serum-free medium containing about 5 × 10^5^ C6-Luc cells were injected into the brain. Bioluminescence imaging and magnetic resonance imaging were used to assess the suitability of the model.

### Fluorescence imaging and pharmacokinetics *in vivo*

After the implementation of the mouse model of orthotopic glioma, DI/Abs or free ICG were injected intravenously in orthotopic glioma-bearing mice (ICG dose = 1 mg/kg). Then, the IVIS Spectrum (PerkinElmer, United States) was used to capture fluorescent images at the baseline, 1 h, 3 h, 6 h, 12 h and 24 h after injection. The mice´s brains and other major organs (heart, liver, spleen, lung, kidney) were obtained for *ex vivo* fluorescence imaging at 24 h post-injection. In addition, to investigate *in vivo* pharmacokinetic of DI/Abs, a single injection of DI/Abs (ICG dose = 1 mg/kg) was intravenously given in Balb/c nude mice (n = 3). At 0.083 h, 0.5 h, 1 h, 2 h, 4 h, 8 h, 12 h,24 h and 48 h, 5 μL of blood was collected via the tail and diluted with EDTA solution (100 μL) in a 96-well plate. The intensity of ICG fluorescence of blood samples was measured and the half-life was calculated after fitting.

### Immunofluorescence staining of *ex vivo* brain tissues

After 24 h from the DI/Abs injection, the mice´s brains were removed, frozen at -80°C, embedded and sliced. Then, cell nuclei were stained with DAPI and monocytes/macrophages were stained with Cy3-labeled F4/80 antibodies. The fluorescence distribution in brain tissues was observed by using fluorescence microscopy.

### Photothermal-chemotherapeutic effect against orthotopic glioma *in vivo*

The glioma-bearing mice were randomly divided into 6 groups (n = 5): (Ⅰ) PBS; (Ⅱ) PBS + laser; (Ⅲ) Dox + ICG + laser; (Ⅳ) Dox/Abs; (Ⅴ) ICG/Abs + laser; (Ⅵ) DI/Abs + laser (ICG = 1 mg/kg, Dox = 0.5 mg/kg, laser wavelength: 808 nm, power density: 1 W/cm^2^). The temperature of the glioma at the baseline, 1 min, 3 min and 5 min was monitored by an infrared thermography camera in groups Ⅱ, Ⅲ, Ⅴ and Ⅵ. Treatments were performed every 3 days, three times in total. Bioluminescence imaging was used to monitor the growth of glioma at days 8, 11, 14 and 17. After the last treatment, mice´s brains were collected and used to evaluate glioma development with H&E staining. In addition, mice were weighed every 3 days to determine the body weight changes. The mice´s survival was continuously monitored.

### Toxicity of DI/Abs *in vivo*

The toxicity of DI/Abs was evaluated in healthy Balb/c mice. There were 3 different treatment groups: group 1 was injected once with physiological saline (control group), group 2 was injected once with DI/Abs and group 3 was injected once every three days with DI/Abs, three times in total (ICG = 1 mg/kg, Dox = 0.5 mg/kg). The liver, lung, heart, kidney, spleen and brain from the three groups were obtained and evaluated with H&E staining. Blood samples were obtained to measure the concentration of alanine aminotransferase, aspartate aminotransferase and blood urea nitrogen at 12 h post-injection.

### Statistical analysis

In each group, we collected no less than three samples. All experimental data were presented as mean ± standard deviation (SD). Statistical significance was evaluated by using the Student's *t*-test. A *p*-value < 0.05 was considered statistically significant.

## Results and Discussion

### Preparation and characterization of DI/Abs

**Figure 2:**
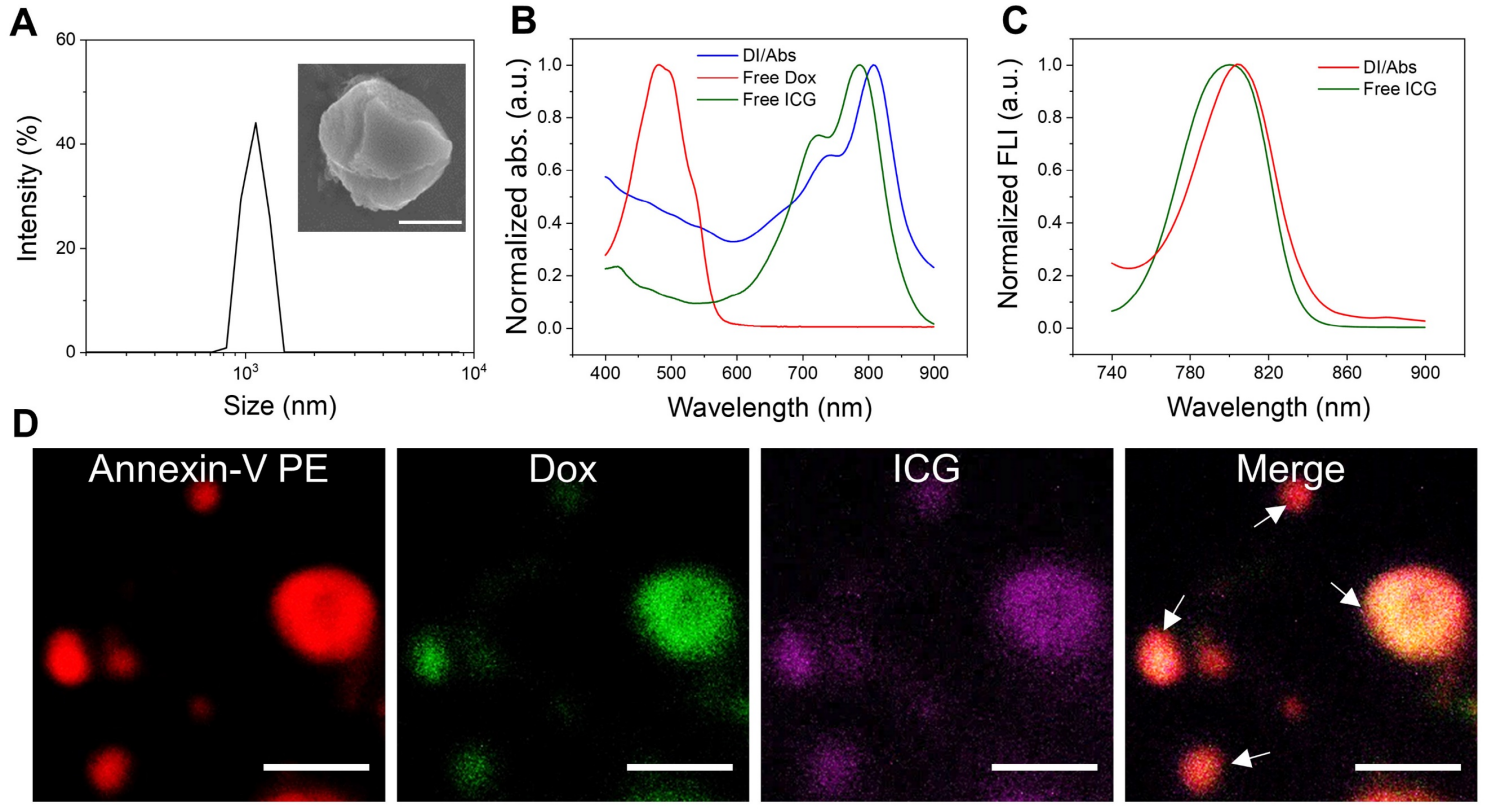
The characterization of DI/Abs. (**A**) The size distribution of DI/Abs. A representative scanning electron microscopic image of single magnified DI/Ab is visualized. Scale bar = 0.5 μm. (**B**) Ultraviolet-visible-near infrared absorption spectra of DI/Abs, free ICG and free Dox. (**C**) Fluorescence spectra of free ICG and DI/Abs. Excitation wavelength = 710 nm. (**D**) Confocal laser scanning microscopic images of DI/Abs stained with Annexin V-PE. The white arrows indicate DI/Abs. Scale bar = 5 μm.

The drug-loaded Abs were obtained by using Dox-treated macrophages, and chemically coupled with ICG to form DI/Abs (Figure S1 and S2). Compared with previous methods, including ultraviolet irradiation, starvation/hypoxia and hydrogen peroxide treatments, our strategy showed the advantages of simplicity, efficiency and no need for additional drug loading processes or special equipment (Table S1). The hydrodynamic size of DI/Abs was measured to be around 1 μm with irregular spherical morphology (Figure 2A and S3). The characteristic absorption bands of Dox and ICG in DI/Abs were detected in the range from 400 nm to 900 nm (Figure 2B). The fluorescence emission spectra were similar to those of free ICG (Figure 2C). These results confirmed that engineered DI/Abs were successfully loaded with Dox and ICG. The confocal laser scanning microscopy (CLSM) analysis further verified the results. The high PS expression on the surface of apoptotic bodies allowed us to specifically label and characterize Abs using annexin V. The fluorescence signal of PS (red) is highly colocalized with the fluorescence signals of Dox (green) and ICG (purple) (Figure 2D). The drug contents of Dox and ICG in a single apoptotic body were measured to be 2.7×10^-6^ μg and 5.5×10^-6^ μg (Figure S4). The photothermal performance of engineered DI/Abs was efficiently evaluated, with the temperature of DI/Abs reaching 65.4℃ within 5 min under laser irradiation (wavelength: 808 nm, power density: 1.0 W/cm^2^) (Figure S5).

### Hitchhiking effect of DI/Abs

**Figure 3:**
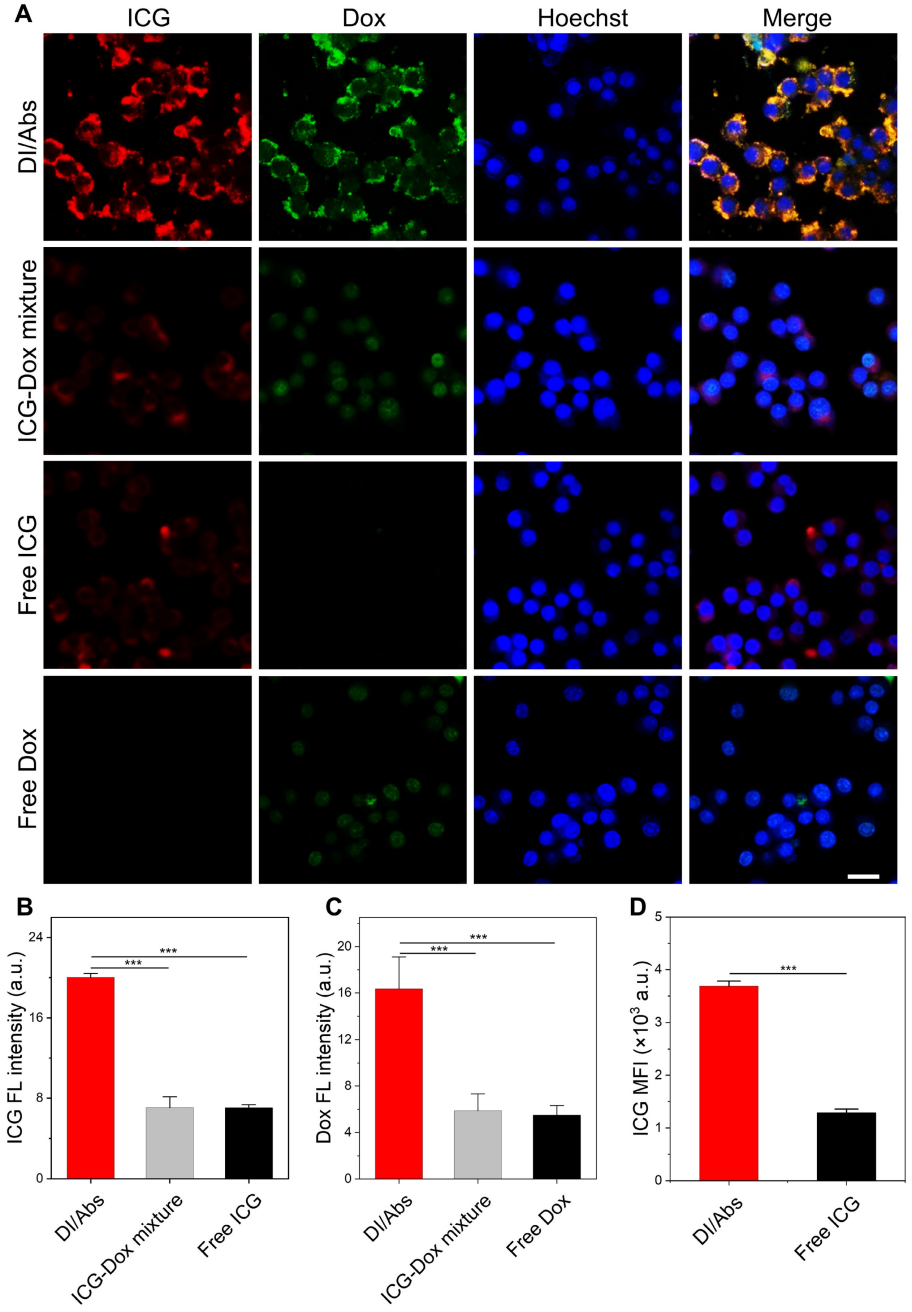
Hitchhiking delivery of DI/Abs. (**A**) Confocal laser scanning microscopic (CLSM) images of Raw 264.7 cells after incubation with DI/Abs, ICG-Dox mixture, free ICG and free Dox for 2 h. Blue: cell nuclei stained with Hoechst 33342. Red: ICG. Green: Dox. Scale bar = 20 μm. Statistical analysis of (**B**) ICG and (**C**) Dox fluorescence intensity from the CLSM images. C_ICG_ = 10 μg/mL, C_Dox_= 5 μg/mL. (**D**) The mean fluorescence intensity of ICG in Raw 264.7 cells incubated with DI/Abs or free ICG for 2 h detected by flow cytometry. C_ICG_ = 10 μg/mL. *** *p* < 0.001.

The Abs were effectively taken up by monocytes/macrophages *in vivo*, showing great potential in tumor-targeted drug delivery through the hitchhiking effect 39. Raw 264.7 cells were selected to simulate the behavior of macrophages *in vivo* due to its safety and easy availability. As shown in Figure 3A, a bright fluorescence signal from ICG (red) and Dox (green) channels was observed after 2 h coincubation of Raw 264.7 cells with DI/Abs. In contrast, a weak fluorescence signal was observed in macrophages treated with ICG-Dox mixture, free ICG and free Dox. The mean fluorescence intensity of ICG and Dox was 2 times higher in the DI/Abs-treated group than in the free ICG- and free Dox-treated groups (Figure 3B, C). Similar results were obtained by flow cytometry (Figure 3D and S6). We demonstrated that Abs were effective carriers of ICG and Dox in Raw 264.7 cells. Dox in DI/Abs did not enter the nucleus of macrophages during the 2 h-treatment period, indicating that DI/Abs could prevent the killing of macrophages and ensure the hitchhiking effect. The cytotoxicity assay showed that Raw 264.7 cells cultured with DI/Abs for 12 h maintained more than 80% cell viability (Figure S7). We further investigated the effect of different ICG concentrations on the uptake of Abs by Raw 264.7 cells. The results showed that ICG fluorescence (purple) in Raw 264.7 cells increased concomitantly with the ICG concentration in DI/Abs. The Dox fluorescence (green) was almost unchanged, suggesting that different concentrations of ICG on the surface of Abs did not affect the uptake by macrophages (Figure S8).

### BBB-crossing ability of DI/Abs *in vitro*

**Figure 4:**
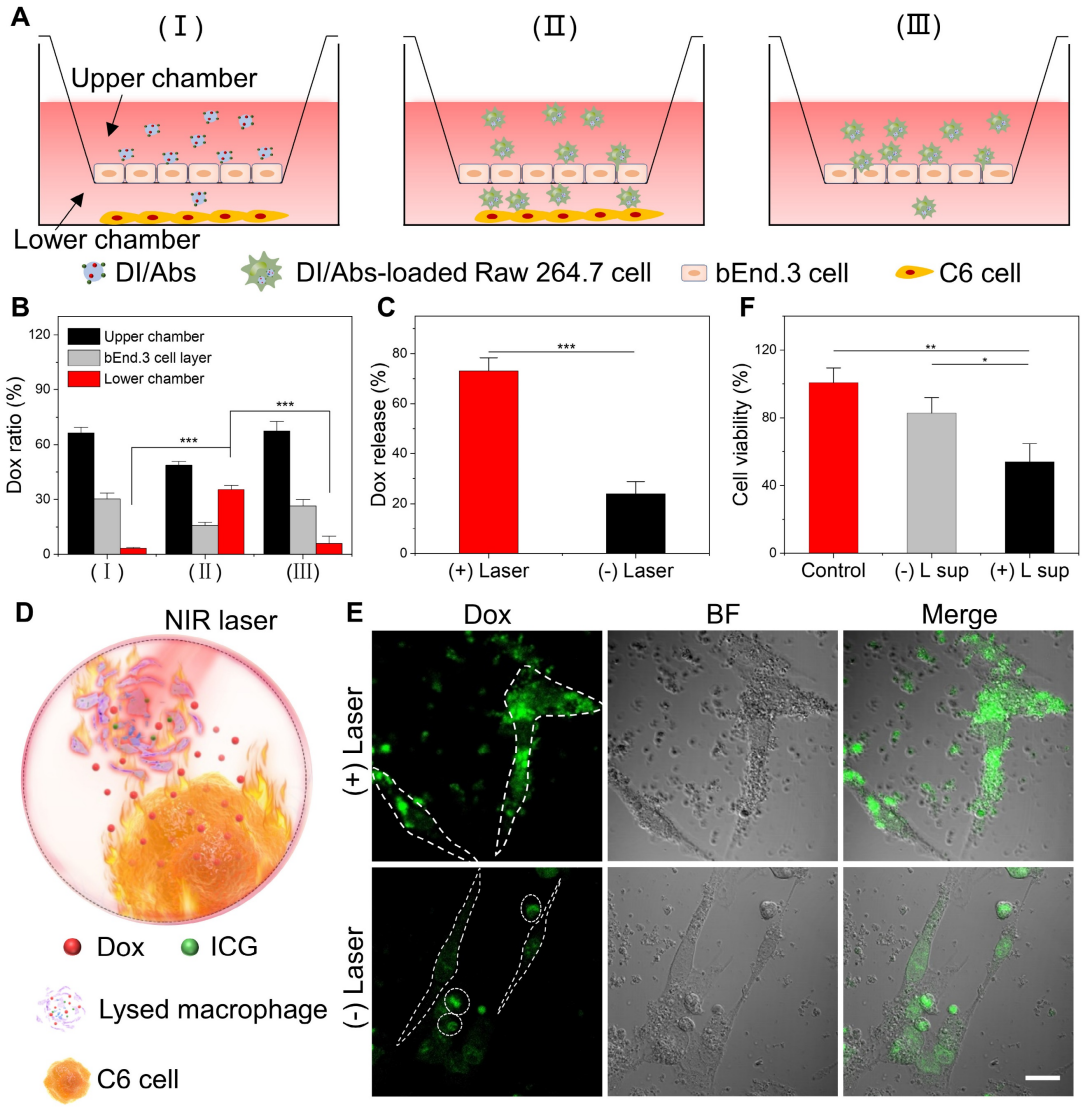
DI/Abs crossed the BBB *in vitro* and photothermal effect promoted Dox release and transport to C6 glioma cells. (**A**) Schematic illustration of the *in vitro* model of BBB evaluating the hitchhiking effect of DI/Abs across the BBB. (**B**) Dox distribution in the transwell system after treatments. (**C**) Dox releasing efficiency of DI/Abs-loaded Raw 264.7 cells with or without laser irradiation (laser wavelength: 808 nm, power density: 1.0 W/cm^2^) for 5 min. (**D**) Schematic illustration of C6 glioma cells co-incubated with DI/Abs-loaded Raw 264.7 cells pretreated with or without laser irradiation (laser wavelength: 808 nm, power density: 1.0 W/cm^2^) for 5 min. (**E**) Confocal laser scanning microscopic images of C6 glioma cells after co-incubation with differently treated DI/Abs-loaded Raw 264.7 cells for 12 h. The fusiform dashed circle represents C6 glioma cells, the circular dashed circle represents Raw 264.7 cells. Scale bar = 20 μm. (**F**) Cell viability of C6 glioma cells after incubation with different supernatants for 24 h. “(+) L sup” represents the supernatant of DI/Abs-loaded Raw 264.7 cells with laser irradiation, “(-) L sup” represents the supernatant of DI/Abs-loaded Raw 264.7 cells without laser irradiation. * *p* < 0.05, ** *p* < 0.01, *** *p* < 0.001.

* In vitro* models were utilized to assess the BBB-crossing ability of DI/Abs (Figure 4A). The experiments were conducted in the following three groups: (Ⅰ) DI/Abs with C6 glioma cells; (Ⅱ) DI/Abs-loaded Raw 264.7 cells with C6 glioma cells; (Ⅲ) DI/Abs-loaded Raw 264.7 cells without C6 glioma cells. After 4 h post-treatment, the content of Dox in the lower chamber was determined to assess the ability of DI/Abs to cross the BBB *in vitro*. As shown in Figure 4B, the percentage of Dox was measured to be around 35% in the lower chamber of group Ⅱ. In group I and group III, the percentage of Dox in the lower chamber was approximately 3% and 6%, respectively. These results indicated that DI/Abs could effectively cross the BBB *in vitro* in the presence of macrophages in the upper chamber and C6 glioma cells in the lower chamber. We hypothesized that macrophages with DI/Abs could recognize homing signals released by C6 glioma cells in the lower chamber, enhancing their ability to cross the BBB. We measured Dox concentration in the upper chamber and bEnd.3 cells, respectively, showing a higher Dox concentration in group Ⅰ (upper chamber: 66%, bEnd.3 cells: 30%) and group Ⅲ (upper chamber: 68%, bEnd.3 cells: 26%) compared to group Ⅱ (upper chamber: 49%, bEnd.3 cells: 16%). DI/Abs remained in the upper chamber or the bEnd.3 endothelial cells in absence of macrophages or C6 glioma cells, resulting in a low efficiency of crossing BBB *in vitro*.

### Photothermal effect promoting Dox release from Raw 264.7 cells and transport to C6 glioma cells

We investigated the photothermal effect on Dox release in Raw 264.7 cells. After 5 min of laser irradiation (wavelength: 808 nm, power density: 1.0 W/cm^2^), the percentage of released Dox in the supernatant of DI/Abs-loaded Raw 264.7 cells was around 73%. In the control group, the percentage of released Dox without laser irradiation was approximately 23% (Figure 4C). Photothermal treatment may induce lysis and apoptosis in Raw 264.7 cells, and both the direct release of Dox from lysed cells and the indirect release of Dox entrapped in apoptotic bodies increased, resulting in a large amount of Dox release. We implemented a co-incubation system with DI/Abs-loaded Raw 264.7 cells and C6 glioma cells to verify whether released Dox entered in C6 glioma cells (Figure 4D). When DI/Abs-loaded Raw 264.7 cells were irradiated by laser (wavelength: 808 nm, power density: 1.0 W/cm^2^) for 5 min and further incubated with C6 glioma cells for 12 h, an intense Dox fluorescence (green) was observed in C6 glioma cells, indicating that released Dox effectively entered in adjacent cancer cells (Figure 4E). The supernatant from laser irradiation-treated DI/Abs-loaded Raw 264.7 cells was collected and cultured with C6 glioma cells for 24 h, showing that the survival rate of C6 glioma cells decreased by 50%. In contrast, more than 80% of C6 cells survived after incubation with the supernatant of DI/Abs-loaded Raw 264.7 cells without laser irradiation (Figure 4F). The calcein-AM and propidium iodide staining showed a strong red fluorescence in C6 glioma cells exposed to the supernatant of laser irradiation-treated DI/Abs-loaded Raw 264.7 cells, confirming the enhanced release of Dox by photothermal effect (Figure S9).

### Fluorescence imaging and pharmacokinetics *in vivo*

**Figure 5:**
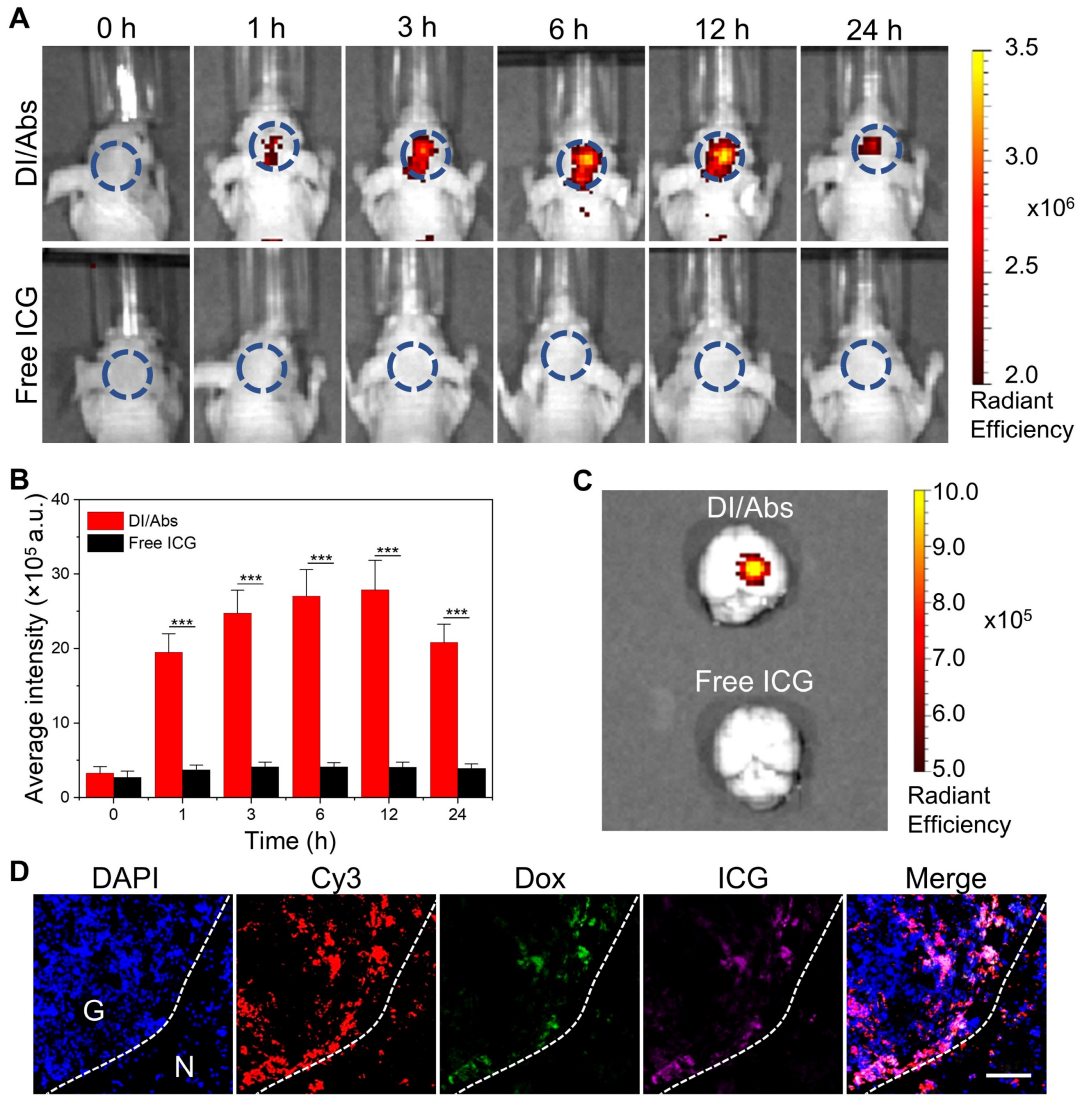
BBB-crossing ability of DI/Abs *in vivo*. (**A**) Real-time fluorescence imaging of glioma-bearing mice after injection with DI/Abs or free ICG at different timepoints. Blue circles show the brain tumor regions. ICG dose = 1 mg/kg. Imaging parameters: Excitation wavelength = 710 nm, Emission wavelength = 800 nm, exposure time = 5 s. (**B**) Quantitative fluorescence analysis in glioma sites at different timepoints. (**C**) Fluorescence images of the *ex vivo* brain from sacrificed mice at 24 h post-injection. Imaging parameters: Excitation wavelength = 710 nm, Emission wavelength = 800 nm, exposure time = 5 s. (**D**) Fluorescence images of a glioma tissue section from sacrificed mice at 24 h after DI/Abs injection. Blue: cell nuclei stained with DAPI. Red: monocytes/macrophages stained with Cy-3 labeled F4/80 antibodies. Green: Dox. Purple: ICG. Scale bar = 100 μm. *** *p* < 0.001.

We further investigated the ability of DI/Abs to cross the BBB and target glioma tumor tissues *in vivo*. An orthotopic glioma model was successfully implemented in Balb/c nude mice using C6-Luc glioma cells (Figure S10). As shown in Figure 5A, the near-infrared fluorescence intensity in the mouse brain increased over time and reached the maximum at 12 h after intravenous injection of DI/Abs (ICG dose = 1 mg/kg). On the contrary, no near-infrared fluorescence was detected in mice treated with free ICG. After 12 h from the injection, the quantitative analysis showed that the fluorescence intensity was approximately six times higher in the DI/Abs-treated group than in the free ICG-treated group (Figure 5B). The signal-to-background ratio of glioma in mice treated with DI/Abs reached 7, exceeding the value reported in previous literature 45, 46. *Ex vivo* fluorescence images showed that the near-infrared fluorescence intensity was high in brain tumor tissues injected with DI/Abs, whereas no fluorescence signal was detected in normal tissues (Figure 5C). For the *in vivo* biodistribution and pharmacokinetics, the fluorescence signals of the DI/Abs injection group were mainly concentrated in the liver and spleen (Figure S11), and the half-life of DI/Abs could reach 0.25 h (Figure S12). The brain tissues of the DI/Abs-treated group were removed and further evaluated with immunofluorescence staining. Consistently with the fluorescence range of Cy3-labeled monocytes/macrophages (red), highly overlapped ICG (purple) and Dox (green) signals were observed in the brain tumor region (Figure 5D). These results demonstrated that DI/Abs crossed the BBB *in vivo* and accumulated in brain tumor regions through the hitchhiking effect.

### The photothermal-chemotherapeutic effect against glioma *in vivo*

**Figure 6:**
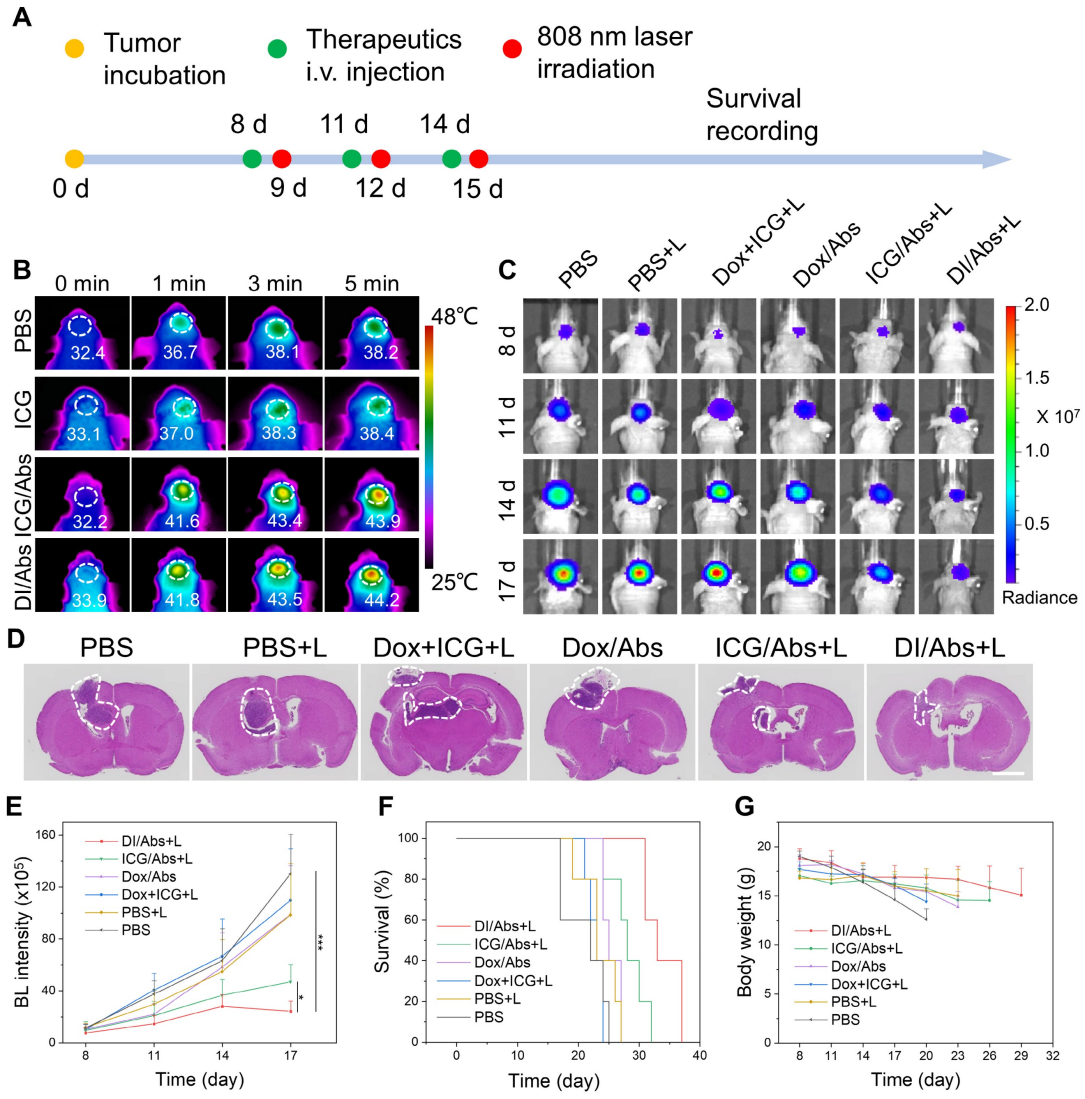
**Photothermal-chemotherapeutic effects of DI/Abs in a mouse model of orthotopic glioma.** (**A**) Schematic illustration of the combined photothermal-chemotherapeutic treatment against glioma. (**B**) Representative infrared thermal images of glioma regions in different treatment groups after irradiation with laser (wavelength: 808 nm, power density: 1.0 W/cm^2^) for 5 min at different timepoints. ICG dose = 1 mg/kg. (**C**) Representative bioluminescence images of the mice brain at different timepoints. (**D**) Hematoxylin and eosin staining of brain sections. Scale bar = 2 mm. (**E**) Quantitative bioluminescent signal intensity of glioma sites at different timepoints. BL represents the abbreviation of bioluminescence. * *p* < 0.05 vs. the ICG/Abs + laser group, *** *p* < 0.001 vs. the PBS group. (**F**) Survival curves of mice for different treatment groups. (**G**) Changes of body weight in different treatments groups. ICG dose = 1 mg/kg. Dox dose = 0.5 mg/kg.

We investigated the potential photothermal-chemotherapeutic effect of DI/Abs in a mouse model of orthotopic glioma (Figure 6A). C6 glioma-bearing mice were divided into the following 6 groups (n = 5 per group): (Ⅰ) phosphate-buffered saline (PBS); (Ⅱ) PBS + laser; (Ⅲ) Dox + ICG + laser; (Ⅳ) Dox/Abs; (Ⅴ) ICG/Abs + laser; (Ⅵ) DI/Abs + laser. After photothermal treatment (laser wavelength: 808 nm, power density: 1.0 W/cm^2^) for 5 min, the temperature of the mouse brain in the V and VI groups was 44.2℃ and 43.9℃, respectively. The temperature exceeded the effective photothermal threshold against cancer (43℃) 47. In the II and III groups, the photothermal temperature increased to approximately 38℃ (Figure 6B and S13). The results indicated that DI/Abs could be an effective photothermal treatment against glioma. We further assessed the overall photothermal-chemotherapeutic effect of DI/Abs by bioluminescence imaging (Figure 6C). In the VI group, the signal intensity in the mouse brain was lower than that in the other groups (Figure 6E), indicating a decreased survival of C6 glioma cells. The results were further confirmed by hematoxylin and eosin (H&E) staining (Figure 6D). The median survival time of mice in the VI group was longer (33 days) than mice treated with chemotherapy alone (25 days, group Ⅳ) or phototherapy alone (28 days, group V) (Figure 6F). These results indicated that a combined photothermal-chemotherapeutic effect inhibited the growth of glioma cells more effectively than single treatments. The weight of mice in the VI group showed no substantial changes compared to the other treatment groups, suggesting an adequate tolerability of the therapy (Figure 6G).

### The toxicity of DI/Abs *in vivo*

**Figure 7:**
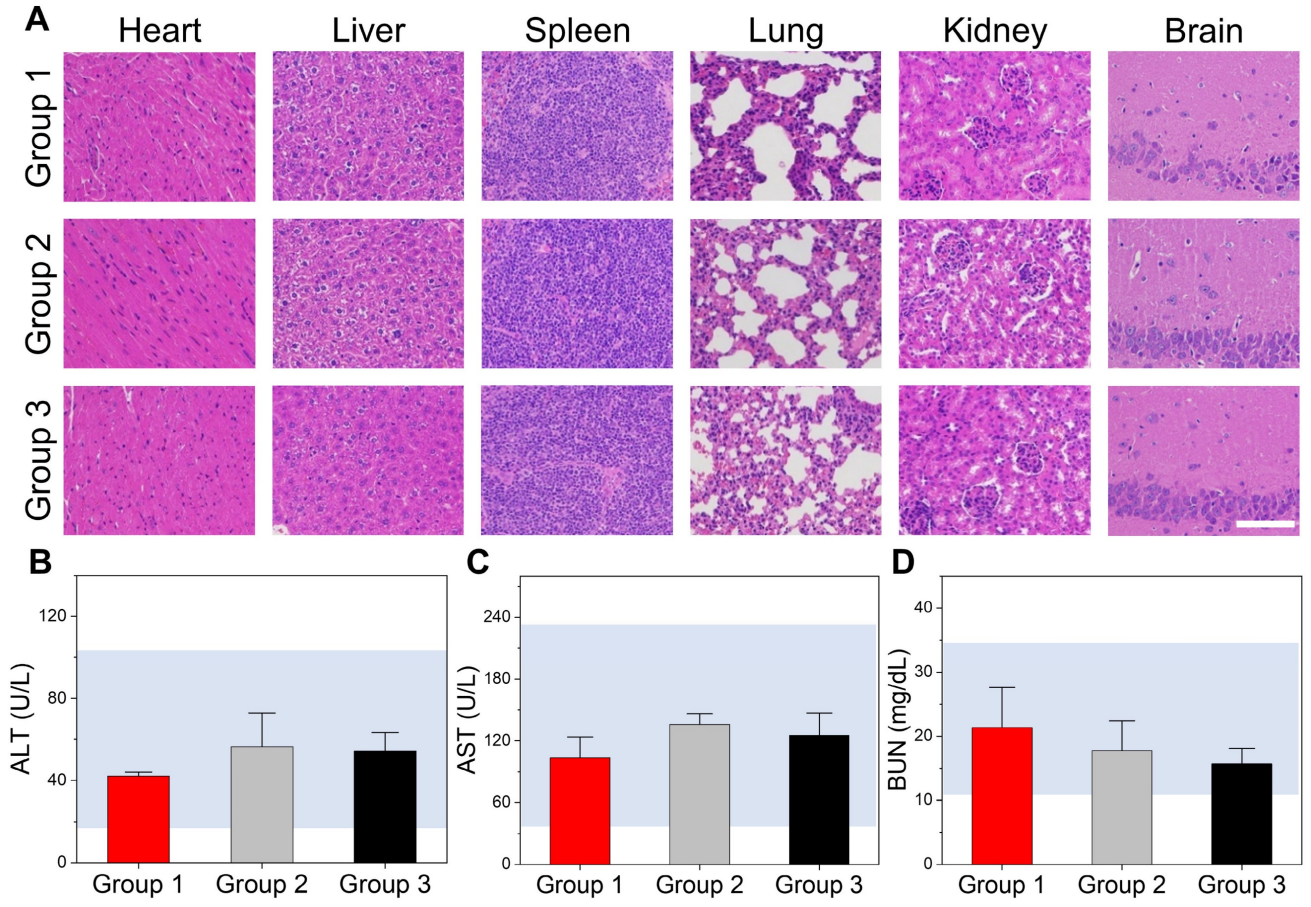
** Toxicity evaluation of DI/Abs *in vivo*.** (**A**) Hematoxylin and eosin staining of different mice tissues (heart, liver, spleen, lung, kidney and brain) in different treatment groups. Scale bar = 100 μm. (**B**) Alanine aminotransferase (ALT), (**C**) aspartate aminotransferase (AST) and (**D**) blood urea nitrogen (BUN) levels in different treatment groups. Mice in the group 1 were injected once with saline. Mice in the group 2 were injected once with DI/Abs. Mice in the group 3 were injected once every three days with DI/Abs, three times in total. ICG dose = 1 mg/kg. Dox dose = 0.5 mg/kg.

Healthy Balb/c mice were divided into three groups to evaluate the acute toxicity of DI/Abs *in vivo*: (Ⅰ) an intravenous injection of physiological saline (control group); (Ⅱ) a single intravenous injection of DI/Abs; (Ⅲ) a single intravenous injection of DI/Abs administered every three days, three times in total (ICG = 1 mg/kg, Dox = 0.5 mg/kg). After 12 h from the last injection, the mice were sacrificed and the major organs were removed for histological examination by H&E staining. No inflammation or tissue damage was detected in all the groups (Figure 7A). We analyzed different blood parameters, including alanine aminotransferase, aspartate aminotransferase and blood urea nitrogen. No abnormal values were found in the II and III groups compared to the control group (Figure 7B-D). These results indicated that our engineered DI/Abs did not cause acute toxicity in mice.

## Conclusions

We established an efficient method to produce engineered DI/Abs loaded with Dox and ICG. The DI/Abs were effectively phagocytosed by macrophages, crossed the BBB *in vitro* and *in vivo* through the hitchhiking effect and exhibited a combined photothermal-chemotherapeutic effect in a mouse model of orthotopic glioma. The median survival time of glioma-bearing mice treated with DI/Abs was significantly longer than that of all the other treatment groups. Our study provided a novel DDS across the BBB which may be used in the future to treat brain tumors or other diseases of the central nervous system.

## Supplementary Material

Supplementary figures and table.

## Abbreviations

BBB: blood-brain barrier
Abs: apoptotic bodies
DDSs: drug delivery systems
PS: phosphatidylserine
Dox: doxorubicin
ICG: indocyanine green
CCK-8: Cell Counting Kit-8
DMEM: Dulbecco's modified Eagle's medium
CLSM: confocal laser scanning microscopy
H&E: hematoxylin and eosin
