# Supplementary material for: Engineered apoptotic bodies hitchhiking across the blood-brain barrier achieved a combined photothermal-chemotherapeutic effect against glioma
Source: Theranostics. 2023 May 15;13(9):2966–78. doi: 10.7150/thno.80632 (PMC10240828; doi:10.7150/thno.80632)
Supplement: Supplementary file 1 — Supplementary figures and table. [file thnov13p2966s1.pdf]

Supporting Information

Engineered apoptotic bodies hitchhiking across the blood-brain barrier achieved a combined photothermal-chemotherapeutic effect against glioma

Yu Liu[1,2,4#], Dehong Hu[1,#], Duyang Gao[1,✉], Ping Gong[3], Hairong Zheng[1], Minjie Sun[2,✉], Zonghai Sheng[1,✉]

[1] Paul C. Lauterbur Research Center for Biomedical Imaging, Institute of Biomedical and Health Engineering, Shenzhen Institute of Advanced Technology, Chinese Academy of Sciences, Shenzhen, 518055, P.R. China

[2] State Key Laboratory of Natural Medicines, Department of Pharmaceutics, China Pharmaceutical University, Nanjing, 210009, P. R. China

[3] Guangdong Key Laboratory of Nanomedicine, CAS Key Laboratory of Health Informatics, Shenzhen Bioactive Materials Engineering Lab for Medicine, Institute of Biomedicine and Biotechnology, Shenzhen Institute of Advanced Technology, Chinese Academy of Sciences, Shenzhen, 518055, P. R. China

[4] Cancer Centre, Institute of Translational Medicine, Faculty of Health Sciences, University of Macau, Taipa, Macau SAR, 999078, China

[#] These authors contributed equally to this work.

✉ Corresponding authors: Email address: dy.gao@siat.ac.cn (D. Gao); msun@cpu.edu.cn (M. Sun); zh.sheng@siat.ac.cn (Z. Sheng).

Supplementary Figures

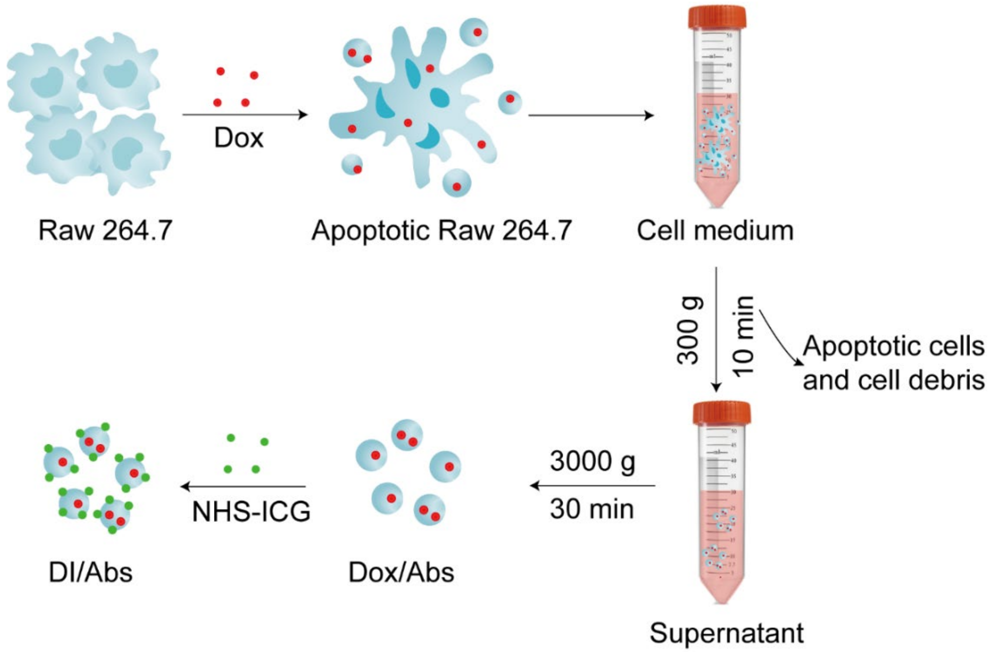

Figure S1. Schematic diagram of the preparation process of DI/Abs.

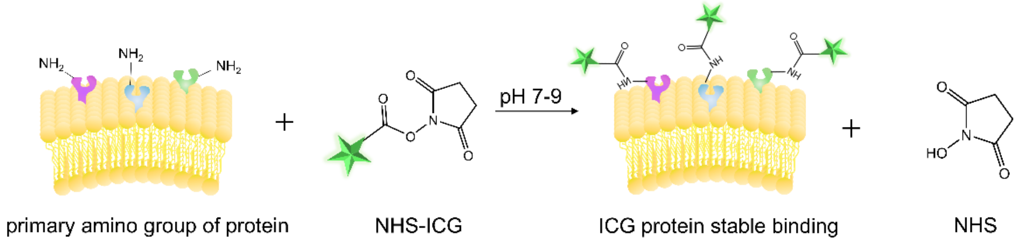

Figure S2. Schematic diagram of the coupling between NHS-ICG and the surface protein of the apoptotic body. The mechanism of NHS-ICG coupling to apoptotic bodies is that N-hydroxysuccinimide ester (NHS) reacts with primary amino (-NH_2) distributed on the membrane protein of apoptotic bodies at room temperature to form amide bonds, and then ICG is labeled to apoptotic bodies.

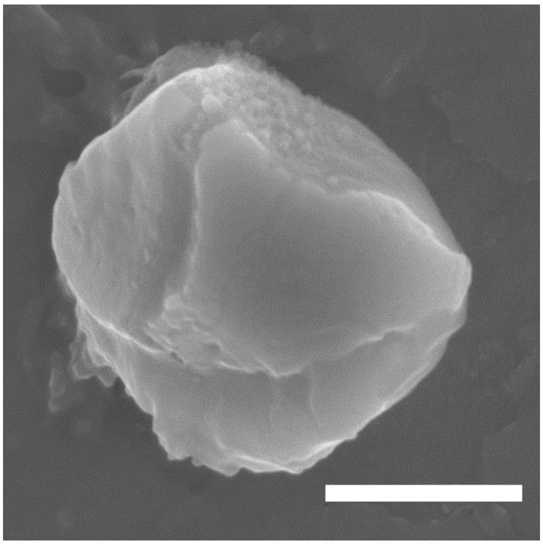

Figure S3. Magnified representative scanning electron microscopic image of DI/Abs. Scale bar = 500 nm.

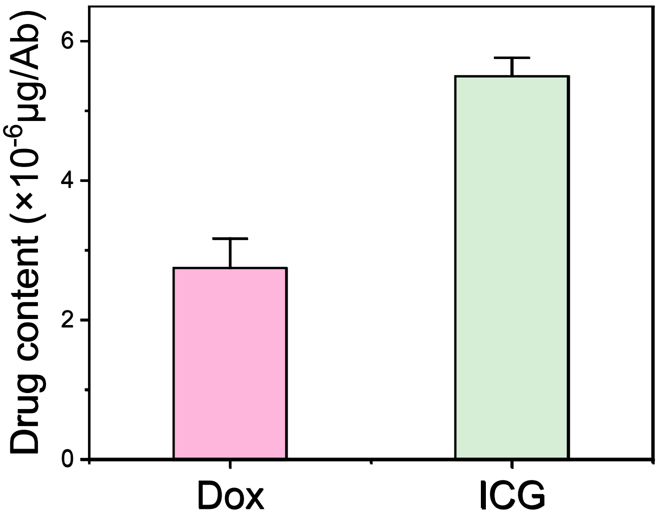

Figure S4. The drug loading content of Dox and ICG in DI/Abs.

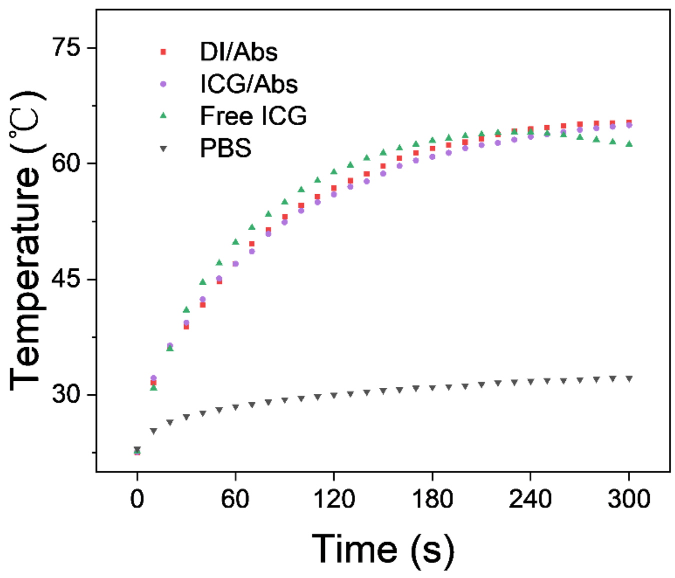

Figure S5. Temperature changes of DI/Abs, ICG/Abs, free ICG and PBS after laser irradiation (wavelength: 808 nm, power density: 1.0 W/cm^2). C_{ICG} = 100 μg/mL.

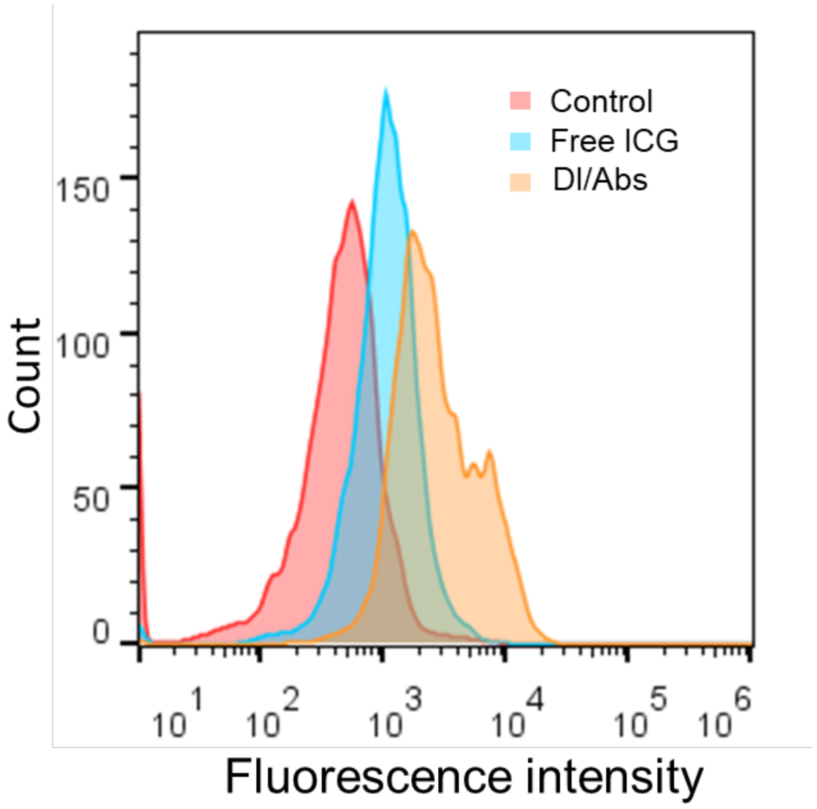

Figure S6. The ICG fluorescence intensity of Raw 264.7 cells incubated with DI/Abs or free ICG for 2 h. C_{ICG} = 10 μg/mL.

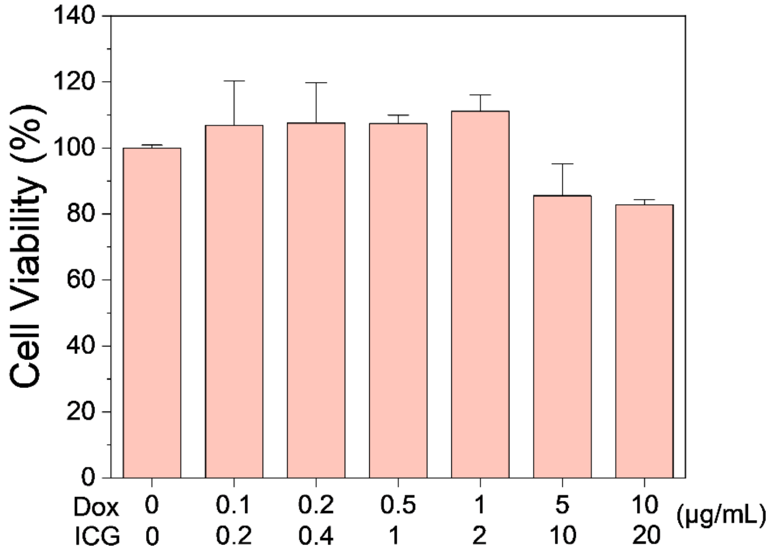

Figure S7. Viability of Raw 264.7 cells after incubation with DI/Abs at different concentrations for 12 h.

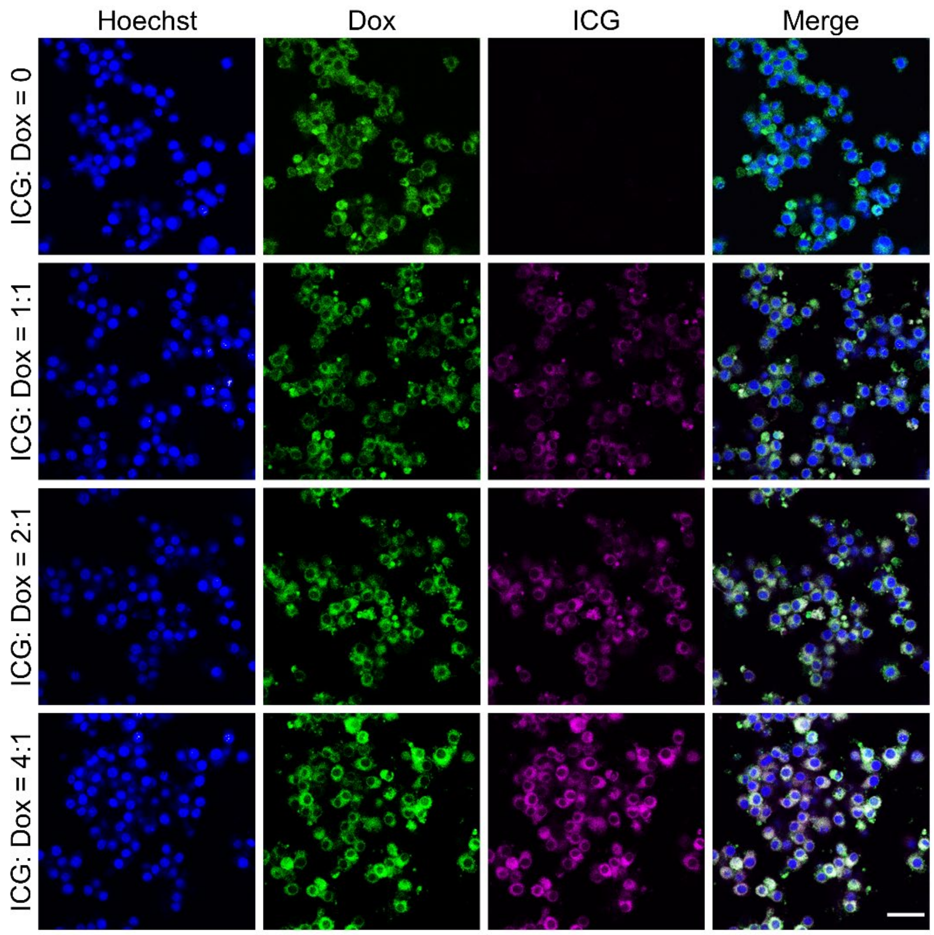

Figure S8. The confocal laser scanning microscopic images of Raw 264.7 cells incubated with different ratios of ICG-modified Abs for 2 h. Blue: cell nuclei stained with Hoechst 33342. Green: Dox. Purple: ICG. C_{Dox} = 5 μg/mL, C_{ICG} = 0, 5, 10 and 20 μg/mL. Scale bar = 40 μm.

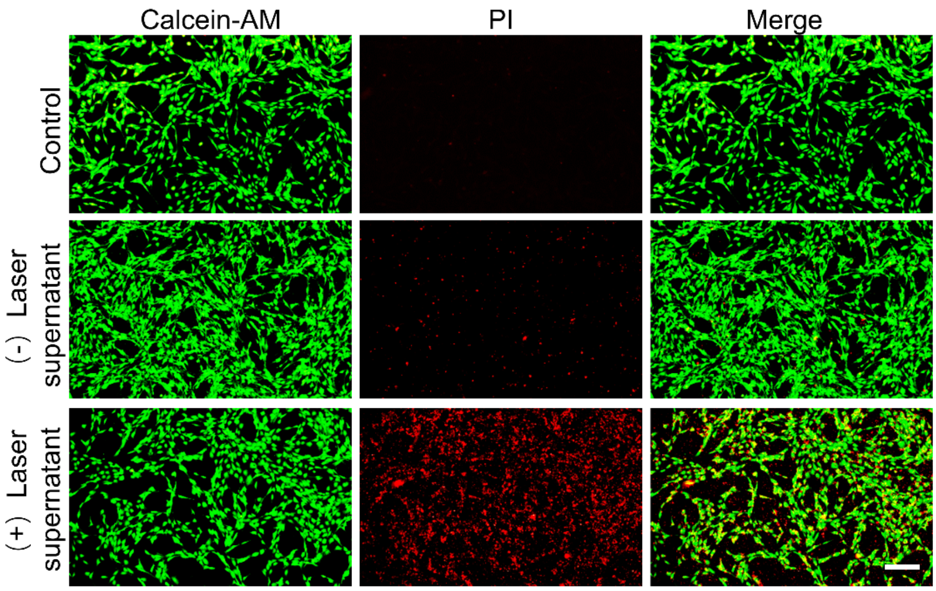

Figure S9. Live/dead staining images of C6 glioma cells incubated with different supernatants for 24 h. Scale bar = 200 μm.

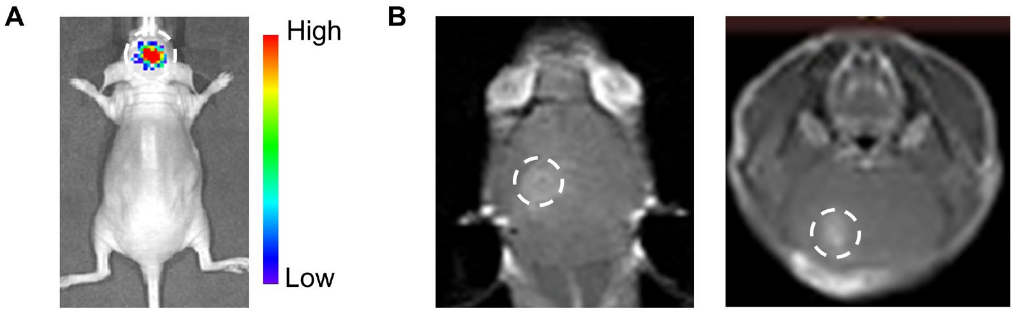

Figure S10. Validation of the mouse model of orthotopic glioma. (**A**) Bioluminescence imaging of an orthotopic glioma-bearing mouse. (**B**) Coronal (left) and transverse (right) magnetic resonance imaging of an orthotopic glioma-bearing mouse. The white circles indicate the glioma region.

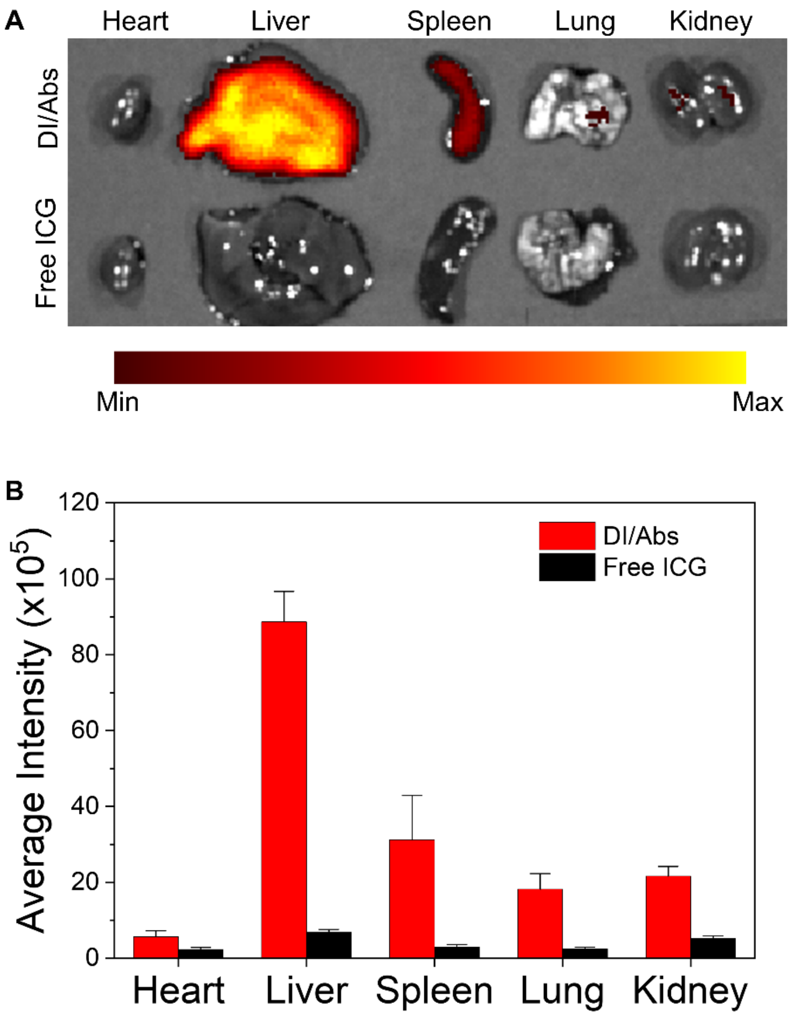

Figure S11. (**A**) *Ex vivo* fluorescent images of major organs at 24 h post-injection. (**B**) Quantitative biodistribution of DI/Abs and free ICG in major organs.

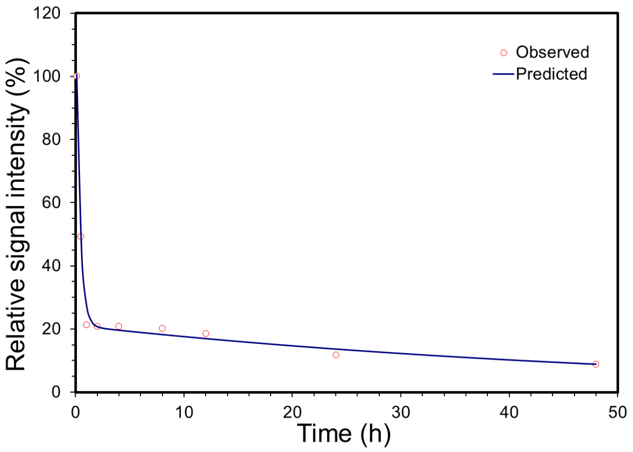

Figure S12. The fitted curve of ICG fluorescence intensity in blood collected at various time intervals.

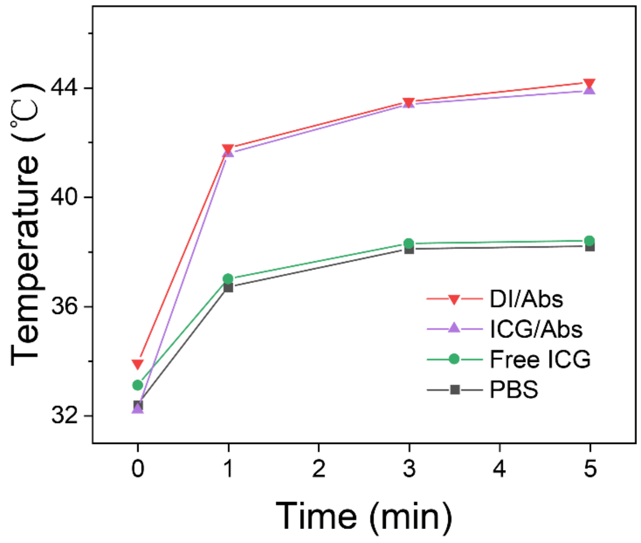

Figure S13. The temperature of glioma after irradiation with laser (wavelength: 808 nm, power density: 1.0 W/cm^2) in different treatment groups.

Table S1. Comparison of the preparation methods for apoptotic bodies.

**Method**	**Speed**	**Yield**	**Additional drug loading process**	**Cell source**	**Application**	**Ref**.
UV induction	Medium	Medium	With	Human MSCs, EL4 cells	Intrauterine adhesions, EL4 subcutaneous tumors	[1, 2]
H_2O_2 treatment	Slow	Medium	With	B16F10 cells	Parkinson's disease	[3]
Staurosporine induction	Fast	High	With	T cells	Cutaneous inflammation and colitis	[4]
PFA+DDT induction	Fast	High	With	4T1 cells	4T1 orthotopic metastasis model	[5]
Starvation induction	Slow	Low	With	Cancer cells	Bacterial infections	[6]
**Chemotherapeutic induction**	**Fast**	**High**	**Without**	**Raw 264.7 cells**	**Orthotopic glioma**	**Current study**

UV: ultraviolet, MSCs: mesenchymal stem cells, PFA: paraformaldehyde, DDT: dithiothreitol.

References

[1] Xin L, Wei C, Tong X, Dai Y, Huang D, Chen J, et al. In situ delivery of apoptotic bodies derived from mesenchymal stem cells via a hyaluronic acid hydrogel: A therapy for intrauterine adhesions. Bioact Mater. 2022; 12: 107-19.

[2] Zheng L, Hu X, Wu H, Mo L, Xie S, Li J, et al. In vivo monocyte/macrophage-hitchhiked intratumoral accumulation of nanomedicines for enhanced tumor therapy. J Am Chem Soc. 2020; 142: 382-91.

[3] Wang Y, Pang J, Wang Q, Yan L, Wang L, Xing Z, et al. Delivering antisense oligonucleotides across the blood-brain barrier by tumor cell-derived small apoptotic bodies. Adv Sci. 2021; 8: 2004929.

[4] Dou G, Tian R, Liu X, Yuan P, Ye Q, Liu J, et al. Chimeric apoptotic bodies functionalized with natural membrane and modular delivery system for inflammation modulation. Sci Adv. 2020; 6: eaba2987.

[5] Zhang K, Fu H, Xing C, Luo Y, Cheng F, Fu Q, et al. "Don't eat me/eat me"-combined apoptotic body analogues for efficient targeted therapy of triple-negative breast cancer. J Mater Chem B. 2021; 9: 8472-79.

[6] Bose RJC, Tharmalingam N, Garcia Marques FJ, Sukumar UK, Natarajan A, Zeng Y, et al. Reconstructed apoptotic bodies as targeted "nano decoys" to treat intracellular bacterial infections within macrophages and cancer cells. ACS Nano. 2020; 14: 5818-35.
